# Nationwide, population-based observational study of the molecular epidemiology and temporal trend of carbapenemase-producing Enterobacterales in Norway, 2015 to 2021

**DOI:** 10.2807/1560-7917.ES.2023.28.27.2200774

**Published:** 2023-07-06

**Authors:** Oskar Ljungquist, Bjørg Haldorsen, Anna Kaarina Pöntinen, Jessin Janice, Ellen Haldis Josefsen, Petter Elstrøm, Oliver Kacelnik, The Norwegian Study Group on CPE, Arnfinn Sundsfjord, Ørjan Samuelsen, Members of The Norwegian Study Group on CPE, Nina Handal, Trond E. Ranheim, Bent-Are Hansen, Andreas F. Mjøen, Paul Christoffer Lindemann, Einar Nilsen, Kyriakos Zaragkoulias, Hege Elisabeth Larsen, Jørgen Vildershøj Bjørnholt, Karianne Wiger Gammelsrud, Aleksandra Jakovljev, Iren Høyland Löhr, Anders Bredberg, Åshild Marvik, Christina Minge, Ståle Tofteland, Kristina Papp, Annette Onken, Einar Tollaksen Weme, Brian Guennigsman

**Affiliations:** 1 Norwegian National Advisory Unit on Detection of Antimicrobial Resistance, Department of Microbiology and Infection Control, University Hospital of North Norway, Tromsø, Norway; 2 Research Group on Host-Microbe Interactions, Department of Medical Biology, UiT The Arctic University of Norway, Tromsø, Norway; 3 Division of Infection Medicine, Department of Clinical Sciences, Lund University, Lund, Sweden; 4 Department of Biostatistics, Faculty of Medicine, University of Oslo, Oslo, Norway; 5 Department of Antibiotic Resistance and Infection Prevention, Norwegian Institute of Public Health, Oslo, Norway; 6 The members forming the Norwegian Study Group on CPE are listed below; 7 Microbial Pharmacology and Population Biology Research Group, Department of Pharmacy, UiT The Arctic University of Norway, Tromsø, Norway

**Keywords:** carbapenemase, Enterobacterales, NDM, OXA-48, molecular epidemiology, whole genome sequencing, high-risk clones

## Abstract

**Introduction:**

National and regional carbapenemase-producing Enterobacterales (CPE) surveillance is essential to understand the burden of antimicrobial resistance, elucidate outbreaks, and develop infection-control or antimicrobial-treatment recommendations.

**Aim:**

This study aimed to describe CPE and their epidemiology in Norway from 2015 to 2021.

**Methods:**

A nationwide, population-based observational study of all verified clinical and carriage CPE isolates submitted to the national reference laboratory was conducted. Isolates were characterised by antimicrobial susceptibility testing, whole genome sequencing (WGS) and basic metadata. Annual CPE incidences were also estimated.

**Results:**

A total of 389 CPE isolates were identified from 332 patients of 63 years median age (range: 0–98). These corresponded to 341 cases, 184 (54%) being male. Between 2015 and 2021, the annual incidence of CPE cases increased from 0.6 to 1.1 per 100,000 person-years. For CPE-isolates with available data on colonisation/infection, 58% (226/389) were associated with colonisation and 38% (149/389) with clinical infections. WGS revealed a predominance of OXA-48-like (51%; 198/389) and NDM (34%; 134/389) carbapenemases in a diversified population of *Escherichia coli* and *Klebsiella pneumoniae*, including high-risk clones also detected globally. Most CPE isolates were travel-related (63%; 245/389). Although local outbreaks and healthcare-associated transmission occurred, no interregional spread was detected. Nevertheless, 18% (70/389) of isolates not directly related to import points towards potentially unidentified transmission routes. A decline in travel-associated cases was observed during the COVID-19 pandemic.

**Conclusions:**

The close-to-doubling of CPE case incidence between 2015 and 2021 was associated with foreign travel and genomic diversity. To limit further transmission and outbreaks, continued screening and monitoring is essential.

**Key public health message**

**What did you want to address in this study?**

Carbapenems are effective antibiotics used to treat serious and multidrug-resistant bacterial infections. Carbapenemase-producing Enterobacterales (CPE) are bacteria that can cause difficult to cure infections and challenge the usefulness of carbapenems. Besides the ability to infect people, CPE can also colonise individuals. We wanted to describe CPE in Norway, their epidemiology in 2015–2021 and characteristics of patients with CPE.

**What have we learnt from this study?**

During the study, 332 patients with CPE were identified. Analyses found ca 58% of patients colonised. Between 2015 and 2021, CPE annual incidence in Norway nearly doubled, partly via travel, with people possibly importing diverse CPE, and partly via local-level CPE transmission between patients in healthcare. For ca 18% of patients with CPE, CPE acquisition could not be linked to import and CPE transmission routes remained unclear.

**What are the implications of your findings for public health?**

The increase of CPE incidence during the study period and the large proportion of CPE identified as colonisation underline that continued vigilance for CPE in Norway is important. Risk assessments and maintained emphasis on screening, surveillance and infection control are needed to limit the further spread of these bacteria, as well as their transmission in hospitals, and their establishment in both the healthcare setting and the community.

## Introduction

Carbapenemase production is a mechanism, which allows bacteria (including Enterobacterales) to develop resistance to carbapenems, a group of antibiotics used to treat serious infections. Worldwide, a dramatic increase in infections caused by carbapenemase-producing Enterobacterales (CPE) has been observed since the early 2000s [1]. This development, which is a concern, is characterised by the occurrence of difficult-to-treat multidrug-resistant (MDR) *Escherichia coli* and *Klebsiella pneumoniae* associated with significant morbidity and mortality [2,3]. Consequently, carbapenem-resistant Enterobacterales constitute a major threat to modern healthcare and are listed at the top of the World Health Organization (WHO) priority pathogen list for discovery, research and development of new antibiotics [4]. Fortunately, the development of new β-lactam-β-lactamase inhibitor combinations and cefiderocol has provided alternative treatment options [5,6].

Carbapenemases can be grouped into Ambler class A (e.g. *Klebsiella pneumoniae* carbapenemase (KPC)), class B (e.g. Verona integron-encoded metallo-β-lactamase (VIM), New Delhi metallo-β-lactamase (NDM) and imipenemase (IMP)) and class D (e.g. oxacillinase-48 (OXA-48)) [7]. The relative proportions of carbapenemases show considerable geographical variation across the globe [8]. The genes encoding carbapenemases are most often carried by mobile genetic elements (e.g. plasmids) that can spread horizontally. For CPE, the dissemination mode is complex and comprises expansion of MDR high-risk clones, epidemic plasmids, and transient associations of carbapenemase genes with a diversity of plasmids and clones [9]. In terms of transmission of CPE, this is mainly impelled by healthcare-associated transmission of a small number of clonal lineages [10].

The prevalence of CPE has been low in the Nordic countries and mainly attributed to imported cases of CPE [11,12]. Nonetheless, as an increased prevalence of CPE in clinical samples throughout Europe has been observed since the early 21^st^ century, we expect a similar development in Norway [13,14]. Thus, it is essential to monitor the national epidemiology of CPE to understand the burden of MDR pathogens as well as to support outbreak surveillance and recommendations for antimicrobial treatment and infection control [15,16].

We have previously described the molecular epidemiology of CPE in Norway during the 2007 to 2014 period [17]. In this study we present essential molecular and epidemiological characteristics of CPE in Norway between 2015 and 2021 including potential inter- and intra-regional transmission.

## Methods

### Study setting and data collection

This is a nationwide, population-based observational study, covering the four health regions (North, Central, West and South-East) of Norway. All confirmed CPE isolates submitted to the Norwegian National Advisory Unit on Detection of Antimicrobial Resistance, which is the national reference laboratory (NRL) of CPE in Norway, during 2015–2021 were included. Private and public clinical microbiology laboratories in Norway are required to submit Enterobacterales to the NRL for confirmation that they are CPE and for surveillance. Submission of isolates is based on the Nordic Committee on Antimicrobial Susceptibility Testing (NordicAST) (www.nordicast.org) CPE algorithm for analysis of carbapenemase-production and includes isolates with a meropenem minimum inhibitory concentration (MIC) of > 0.125 mg/L or disc diffusion zone diameter of < 27 mm (2015–2017)/ < 28 mm (2018–2021). Criteria for CPE screening are described in a national infection control guidance document (https://www.fhi.no/nettpub/smittevernveilederen/) and include (i) screening of patients admitted to healthcare institutions outside the Nordic countries or in institutions in Norway or Nordic countries with an ongoing outbreak in the past 12 months, (ii) previous identification of CPE or living with a person with identified CPE infection/carriage, and (iii) admission to departments (e.g. intensive care unit (ICU), burn-units etc.) with particularly vulnerable patients after local considerations. After CPE-verification, the Norwegian Surveillance System for Communicable Diseases (MSIS) and the referring laboratory are notified, generating a request to the responsible physician to provide clinical patient data to MSIS. For this study, clinical data regarding age, sex (female, male, unknown), foreign travel and clinical infection/screening were retrieved from laboratory requisition forms and MSIS.

In accordance with our previous publication, multiple isolates from the same patient were included if they were (i) of different species, (ii) the same species, but harboured a different carbapenemase gene or sequence type (ST), (iii) or if the isolates were of the same species, ST and harboured the same carbapenemase gene, but were identified more than 1 year apart [17]. A case was defined as a patient positive for one or more CPE within 12 months according to criteria set up by MSIS [18]. Thus, the same patient could represent two cases if the CPE isolate was detected > 12 months from the previous last CPE isolate.

### Phenotypic analysis

Species identification was performed using matrix-assisted laser desorption/ionization-time of flight mass spectrometry (MALDI-TOF MS; Bruker Daltonik GmbH, Bremen, Germany) in combination with whole genome sequencing (WGS). Antimicrobial susceptibility testing (AST) was performed by broth microdilution (BMD) using in-house designed premade Sensititre microtitre plates (TREK Diagnostic Systems/Thermo Fisher Scientific, East Grinstead, United Kingdom). Interpretation was based on European Committee on Antimicrobial Susceptibility Testing (EUCAST) clinical MIC breakpoints version 13.0 [19]. MIC data for ceftazidime–avibactam were acquired from 2017 to 2021.

### Molecular and genomic analysis

The presence of carbapenemase genes was initially determined by PCR for *bla*_KPC_, *bla*_IMI_, *bla*_VIM_, *bla*_NDM_, *bla*_IMP_, *bla*_GIM_, *bla*_SPM_, *bla*_SIM_ and *bla*_OXA-48-like_ [20-23]. Genomic DNA sequencing was performed using Illumina and 2×151 bp paired-end. Sequences were assembled using SPAdes v.3.12.0 [24] with contigs shorter than 200 bp and with less than 2× coverage removed. Multilocus sequence types (MLSTs) were retrieved using mlst v.2.19.0 (https://github.com/tseemann/mlst) by comparing the Illumina sequence data to the PubMLST database [25]. The presence of antimicrobial resistance determinants was screened from the assemblies by using AMRFinderPlus v.3.10.1 [26] with minimum of 90% coverage and 90% identity and a secondary 60% identity and 60% coverage cut-off. Additional genome-specific analyses were run on *Klebsiella* genus using Kleborate v.2.0.4 [27]. Further species-specific population structures of *E. coli* and *K. pneumoniae* assemblies were annotated using Prokka v.1.14.6 [28]. Pangenome was estimated by using Panaroo v.1.2.3 [29] with core threshold of 99%, removing invalid genes and merging paralogues. Maximum-likelihood phylogeny was inferred on the core genome alignment by using RAxML v.8.2.12 with the general time reversible (GTR) + Gamma rate model [30]. Phylogenies aligned with major STs and carbapenem resistance mechanisms harboured by the isolates were illustrated using R v.4.1.3 [31]. Microreact web application [32] was used to visualise phylogenies and metadata, including temporal and geographical data, of *E. coli* and *K. pneumoniae*. Ridom SeqSphere+ software (Ridom GmbH, Münster, Germany) was also used to analyse the CPE population. 

### Statistical analysis

Categorical data were summarised as proportions. Incidence rates were determined by dividing the number of annual CPE cases with the population of Norway. For categorical variables, the chi-squared test was used to detect statistical significance. A p value < 0.05 was considered statistically significant. Population data were accessed from Statistics Norway [33].

## Results

### Carbapenemase-producing Enterobacterales’ incidence during 2015–2021

**Figure 1:**
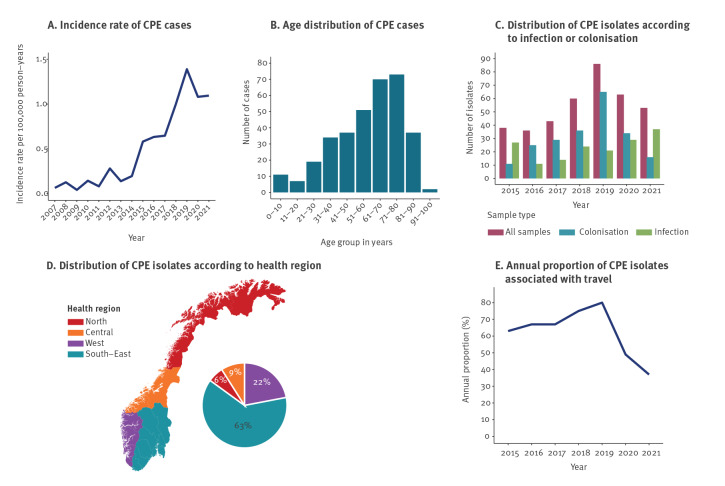
Distributions of CPE cases’ (A) annual incidence rate per 100,000 person-years^a^ and (B) age categories, and distributions of CPE isolates according to (C) infection, colonisation, or (D) health region, as well as (E) annual proportion of CPE isolates associated with travel^b^, Norway 2015–2021^a^ (n = 332 patients representing 341 cases; n = 389 isolates)

In total, 341 CPE cases in 332 patients were identified. The incidence of CPE cases increased between 2015 to 2021, from 0.6 to 1.1 per 100,000 person-years with a peak of 1.4 in 2019 (Figure 1A). The incidence also markedly increased compared with previous surveillance period (2007–2014) when the incidence ranged from 0.04 to 0.28 per 100,000 person-years [17]. The mean CPE incidence between the periods increased from 0.13 in 2007–2014 to 0.91 in 2015–2021.

CPE: carbapenemase-producing Enterobacterales.

^a^ For the incidence rate of CPE cases per 100,000 person-years (panel A), the graph also shows previously published data for 2007–2014 [17].

^b^ The denominator for the annual proportions of CPE isolates related to travel are the total number of CPE isolates in each year.

### Carbapenemase-producing Enterobacterales isolates and patient characteristics

The median age of patients at first identification of CPE was 63 years (range: 0–98 years), and 54% (184/341) of the cases were men. Categorised into age groups, most cases were found in patients aged 61–70 (n = 70; 21%) and 71–80 years (n = 73; 21%) (Figure 1B). In total, 389 CPE isolates were identified during the study period. These are described in Supplementary Table A1. Of these, 226 (58%; 226/389) and 149 (38%; 149/389) were associated with colonisation and infection, respectively, for 14 (4%; 14/389) this information was missing (Figure 1C). Infection-associated CPE were isolated from urine (24%; 94/389), blood (2%; 9/389) and other sample sites (e.g. sputum, skin/wound; 46/389; 12%) and increased in 2020–2021. CPE were identified in all four health regions and at all clinical microbiology laboratories (n = 21) in Norway (Figure 1D). Most CPE isolates (285/389; 73%) were from patients admitted to hospital, whereas 23% (89/389) were obtained from outpatient settings, and 1% (2/389) from long-term care facilities. For 3% (13/389) of isolates this information was missing. Forty-seven patients were positive for more than one CPE isolate.

### Association with travel or hospitalisation abroad

Most isolates (63%; 245/389), were associated with travel and/or hospitalisation abroad. Eighteen percent (70/389) were not associated with import and for 19% (74/389) no information was available. The most common continent of travel was Asia (62%; 152/245), followed by Europe (28%; 68/245), Africa (8%; 19/245) and North/South America (2%; 6/245). Travel to five countries (Spain, India, Pakistan, Thailand and Türkiye – listed from highest to lowest abundance of isolates) represented 56% (137/245) of the isolates associated with travel. The annual proportion of CPE isolates with known travel association increased from 2015 (63%; 24/38) to 2019 (80%; 69/86) but decreased during the COVID-19-pandemic to 49% (31/63) in 2020 and 37% (23/63) in 2021 (Figure 1E). Consequently, the proportion of non-import isolates increased from 14% (30/221) during 2015–2019 to 43% (40/94) during 2020–2021, excluding isolates with no information available.

Rectal screening was the dominant source material for import associated isolates (71%; 174/245), while for non-import isolates, urine was the most common sample material (50%; 35/70). Non-import isolates were identified in all health regions of Norway.

### Bacterial species and carbapenemase distributions

**Figure 2:**
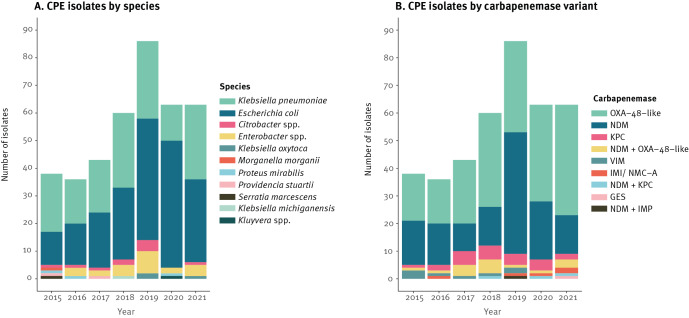
Distribution of isolates of carbapenemase-producing Enterobacterales (A) according to species and (B) carbapenemase variant, Norway 2015–2021 (n = 389)

GES: Guiana extended-spectrum; IMI/NMC-A: imipenem-hydrolysing β-lactamase/not-metalloenzyme-carbapenemase A; IMP: imipenemase; KPC: *Klebsiella pneumoniae* carbapenemase; NDM: New Delhi metallo-β-lactamase; OXA-48: oxacillinase-48; VIM: Verona integron-encoded metallo-β-lactamase.

The CPE population was predominantly *E. coli* (50%; 193/389) and *K. pneumoniae* species complex (*K. pneumoniae* 38% (146/389), *K. quasipneumoniae* subsp. *similipneumoniae* 1% (3/389), *K. quasipneumoniae* subsp. *quasipneumoniae* 0% (1/389) and *K. quasivariicola* 0% (1/389)) and these are listed in Supplementary Table A1 (Figure 2A). Other Enterobacterales species included *Enterobacter* spp. (6%; 23/389), *Citrobacter* spp. (3%; 10/389), *K. oxytoca* (1%; 3/389), *Proteus mirabilis* (1%; 3/389), *Providencia stuartii* (1%; 2/389) and single isolates of *Klebsiella michiganensis, Serratia marcescens, Morganella morganii,* and *Kluyvera* spp.

The carbapenemase gene distributions are shown in Supplementary Table A1 and were *bla*_OXA-48-like_ (51%; 198/389) and *bla*_NDM_ (34%; 134/389) followed by *bla*_KPC_ (6%; 23/389), *bla*_VIM_ (2%; 8/389) and *bla*_IMI/NMC-A_ (1%; 5/389) (Figure 2B). One isolate harboured *bla*_GES-9_. In addition, 20 isolates carried two carbapenemase genes, including *bla*_NDM_ and *bla*_OXA-48-like_ (4%; 16/389), *bla*_NDM_ and *bla*_KPC_ (1%; 3/389) and *bla*_NDM_ and *bla*_IMP_ (0%; 1/389). Eighteen of these were linked to travel and for two, travel information was unavailable.

### Antimicrobial susceptibility and resistance genes

**Figure 3:**
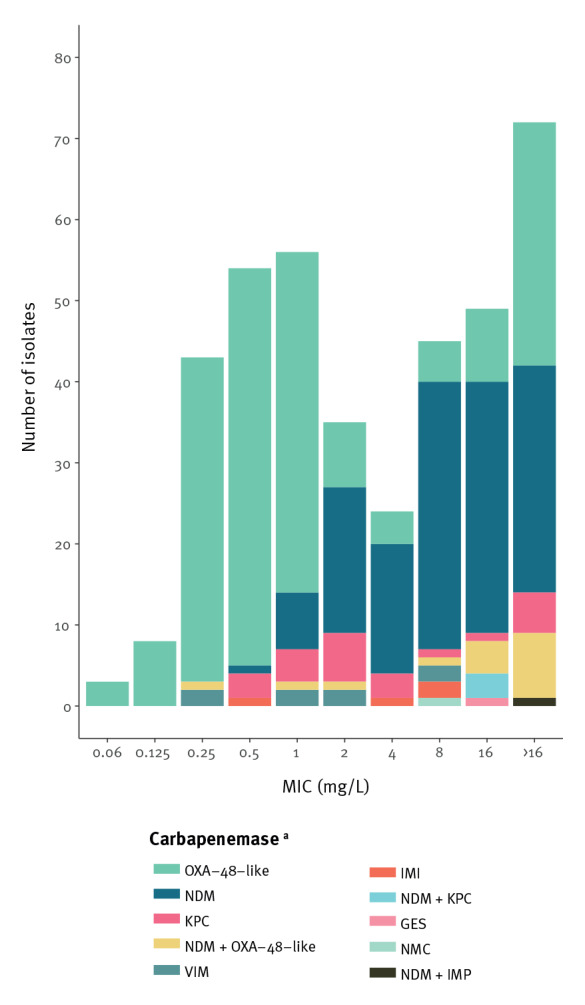
Meropenem MIC distribution of CPE isolates according to carbapenemase variant, Norway, 2015–2021 (n = 389)

Colistin was the only antibiotic with a resistance rate of less than 10% (7%; 25/382) for the whole collection (Table). Eight isolates were found to harbour the plasmid-mediated mobile colistin resistance (*mcr*) genes, *mcr-4.3* (n = 1), *mcr-9.1* (n = 6) or *mcr-9.2* (n = 1). All isolates were susceptible to colistin, as detailed in Supplementary Table A1. For carbapenems, resistance rates were 31% (121/389) for meropenem, 38% (147/389) for imipenem and 95% (369/389) for ertapenem. Eleven isolates (3%; 11/389) had a meropenem MIC lower than the current meropenem screening breakpoint (MIC > 0.125 mg/L), all OXA-48-producers (*bla*_OXA-48_ n = 3; *bla*_OXA-181_ n = 1; and *bla*_OXA-244_ n = 7) (Figure 3). Thirty-nine percent (121/308) of the tested isolates were resistant to ceftazidime–avibactam, all harbouring NDM or other metallo-β-lactamases. One *E. coli* isolate with *bla*_NDM-5_ + *bla*_OXA-232_ was susceptible to ceftazidime–avibactam, which was confirmed upon re-testing. Lack of expression of *bla*_NDM-5_ was confirmed with an immunochromatographic test. All isolates with only class A or class D carbapenemases were susceptible to ceftazidime–avibactam. Simultaneous carriage of extended-spectrum β-lactamase (ESBL)s and other broad-spectrum β-lactamases was common, as shown in Supplementary Table A1. *Bla*_CTX-M_ was found in 57% (77/134) of NDM and 73% (145/198) of OXA-48-like positive isolates contributing to high levels of resistance to aztreonam and cephalosporins. We observed significantly more *bla*_CTX-M_ in the OXA-48 group positive isolates compared with NDM, KPC and VIM (p = 0.003, p = 0.003 and p = 0.0002 respectively). The distribution of *bla*_CTX-M_ with respect to carbapenemase variant is described in Supplementary Table A2. Various 16S rRNA methylase genes leading to high-level broad-spectrum aminoglycoside resistance were identified in 76 isolates (20%; 76/389) of which 74% (56/76) harboured NDM or NDM plus another carbapenemase (Supplementary Table A1).

**Table:** Proportion of isolates resistant to the antibiotics listed, among the dominant species/carbapenemase-variants, and among CPE isolates overall, Norway, 2015–2021 (n = 389)

Antibiotic	*Escherichia coli* OXA-48-like^a^			*Escherichia coli* NDM^a^			*Klebsiella* spp. OXA-48-like^a^			*Klebsiella* spp. NDM^a^			Overall		
	R^b^	N^c^	%	R^b^	N^c^	%	R^b^	N^c^	%	R^b^	N^c^	%	R^b^	N^c^	%
Temocillin	105	108	97	75	77	97	85	85	100	37	38	97	334	351^d^	95
Piperacillin–tazobactam^e^	108	108	100	77	77	100	85	85	100	38	38	100	384	389	99
Cefotaxime	89	108	82	77	77	100	74	85	87	38	38	100	351	389	90
Ceftazidime	52	108	48	76	77	99	74	85	87	38	38	100	311	389	80
Ceftazidime–avibactam^f^	0	96	0	62	62	100	0	64	0	38	38	100	121	308	39
Aztreonam	79	108	73	53	77	69	73	85	86	34	38	89	300	389	77
Meropenem	2	108	2	38	77	50	36	85	42	16	38	42	121	389	31
Imipenem	3	108	3	47	77	61	24	85	28	28	38	74	147	389	38
Ertapenem	94	108	87	77	77	100	84	85	99	38	38	100	369	389	95
Ciprofloxacin	50	108	46	69	77	90	77	85	91	35	38	92	300	389	77
Amikacin	2	108	2	26	77	34	24	85	28	22	38	58	112	389	29
Gentamicin	30	108	28	34	77	44	56	85	66	23	38	61	184	389	47
Tobramycin	92	108	85	46	77	60	67	85	79	38	38	100	241	389	62
Colistin^g^	0	108	0	0	77	0	11	85	13	2	38	5	25	382	7
Fosfomycin^h^	1	108	1	3	76	4	33	85	39	4	38	11	63	388^i^	16
Trimethoprim–sulfamethoxazole	70	108	65	66	77	86	65	85	76	29	38	76	287	389	74

CPE: carbapenemase-producing Enterobacterales; EUCAST: European Committee on Antimicrobial Susceptibility Testing; NDM: New Delhi metallo-β-lactamase; OXA-48: oxacillinase-48.

^a^ Includes isolates with single carbapenemase. Double carbapenemase-producers are excluded.

^b^ Number of resistant isolates according to EUCAST Clinical Breakpoint Tables v. 13.0 [19].

^c^ Denominator.

^d^ Includes *Escherichia coli*, *Klebsiella* spp. (except *K. aerogenes*) and *Proteus mirabilis*.

^e^ Minimum inhibitory concentration values in the area of technical uncertainty (ATU) for piperacillin–tazobactam is interpreted as resistant.

^f^ Data for ceftazidime–avibactam was only obtained in the period 2017–2021.

^g^ Species considered inherently resistant to colistin excluded.

^h^ Susceptibility testing of fosfomycin using broth microdilution should be interpreted with caution since agar dilution is used in the reference method. The EUCAST intravenous (i.v.) breakpoint for fosfomycin is used for interpretation.

^i^ There are 388 isolates listed instead of 389 because one isolate did not give reproducible values.

MIC: minimum inhibitory concentration.

^a^ CPE: carbapenemase-producing Enterobacterales; GES: Guiana extended-spectrum; IMI: imipenem-hydrolysing β-lactamase; NMC-A: not metalloenzyme-carbapenemase A; IMP: imipenemase; KPC: *Klebsiella pneumoniae* carbapenemase; NDM: New Delhi metallo-β-lactamase; OXA-48: oxacillinase-48; VIM: Verona integron-encoded metallo-β-lactamase.

### The population structure of carbapenemase-producing Enterobacterales in Norway

**Figure 4:**
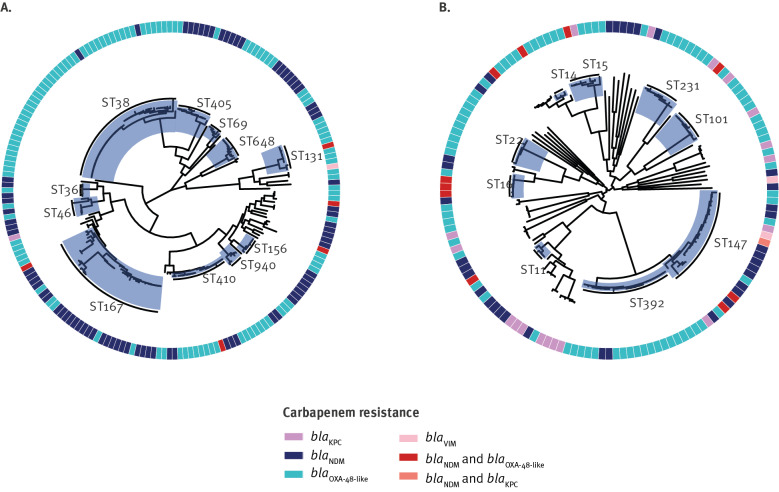
Maximum-likelihood phylogenetic trees of carbapenemase-producing (A) *E. coli* (n = 193) and (B) *K. pneumoniae* (n = 146), Norway, 2015–2021

The carbapenemase-producing *E. coli* population (n = 193) was represented by 50 different STs (Simpson diversity index 91.6%) (Figure 4A, Supplementary Table A1), but dominated by five well known, globally disseminated extraintestinal pathogenic *E. coli* (ExPEC) high-risk clones: ST38 (23%; 44/193), ST167 (11%; 22/193), ST410 (10%; 20/193), ST405 (6%; 11/193), and ST648 (5%; 10/193) which represented > 50% of the isolates [34]. *E. coli* ST38 was primarily harbouring *bla*_OXA-244_ (n = 26) and *bla*_OXA-48_ (n = 15). *E. coli* ST410 was also frequently identified with OXA-48-like carbapenemases, which was dominated by *bla*_OXA-181_ (n = 15). OXA-48-like carbapenemases were also harboured by other ExPEC high-risk clones including ST131 and ST69. The emerging carbapenemase NDM-5 was detected in 37% (72/193) of the *E. coli* isolates in a diverse set of genetic backgrounds (27 different STs), but particularly found in conjunction with the ExPEC clones ST167, ST405 and ST648.

*E. coli*: *Escherichia coli*; *K. pneumoniae*: *Klebsiella pneumoniae*; ST: sequence types.

The STs occurring in largest numbers (n ≥ 5) are highlighted in blue and carbapenem resistance mechanisms are coloured as indicated in the legend. The phylogenetic trees and associated data can be accessed in Microreact projects https://microreact.org/project/dBmEC1d5GNNxN5rqoiTHAe-e-coli-molecular-epidemiology-and-temporal-trend-of-carbapenemase-producing-enterobacterales-in-norway-2015-to-2021-n-193 and https://microreact.org/project/ojCiMw4c9qLGpj5sKpTEsN-k-pneumoniae-molecular-epidemiology-and-temporal-trend-of-carbapenemase-producing-enterobacterales-in-norway-2015-to-2021-n-146.

The carbapenemase-producing *K. pneumoniae* species complex population revealed 50 STs among 151 isolates (Simpson diversity index: 94.6%) (Figure 4B; Supplementary Table A1). The two dominant STs were ST147 (15%; 22/151) and ST392 (14%; 21/151). ST147 harboured a diversity of carbapenemase genes including *bla*_NDM-1_ (n = 11), *bla*_OXA-232_ (n = 4), *bla*_KPC-2_ (n = 2), *bla*_OXA-181_ (n = 1), *bla*_OXA-48_ (n = 1), *bla*_KPC-2_ + *bla*_NDM-1_ (n = 1), *bla*_OXA-232_ + *bla*_NDM-5_ (n = 1), and *bla*_NDM-5_ + *bla*_OXA-232_ (n = 1) and appeared to coincide mainly with import (73%; 16/22) from various countries. In contrast, ST392 isolates were frequently found with *bla*_OXA-48_ (91%; 19/21) and import from Spain (62%; 13/21). Several other STs including other known *K. pneumoniae* MDR high-risk clones like ST11, ST14, ST15, ST101, ST231, ST258 and ST307 were found at similar proportions (2.7–5.3%) [35]. Some STs were only found to harbour one carbapenemase variant like ST231, with all eight ST231 isolates carrying *bla*_OXA-232_ and found in patients with a known travel history to the Indian subcontinent.

A high genomic diversity was also observed for other Enterobacterales species (Supplementary Table A1) and no indication of clonal spread. Fourteen different STs were found among the 19 *Enterobacter* spp. isolates with a defined ST (4 isolates non-typable). ST114 was found in three isolates and two isolates each belonged to ST171, ST418 and ST78. Among *Citrobacter* spp. isolates (n = 10) none of the isolates were of the same ST.

### Outbreaks and onward transmission

The genomic data revealed two outbreaks (with 19 cases overall) and cases of onward transmission (including a total of 9 cases). Twelve *E. coli* ST38-*bla*_OXA-244_ isolates represented a clonal intraregional outbreak involving three hospitals in the western region in 2020 [36]. There were no indications that the index case had acquired the outbreak strain from abroad. Six cases were related to infection. Excluding the isolates linked to the outbreak, only 28% (9/32) of the ST38 isolates were associated with import compared with 63% (245/389) overall. However, no clear genetic and epidemiological link could be established between ST38 isolates where ‘no import’ was reported, as well as no link to and between ST38 isolates related to import or with missing import information. Another, single hospital clonal outbreak of *K. pneumoniae* ST22 *bla*_OXA-181_ (n = 7) was identified in south-east Norway in 2021. Also, in this instance, there was no clear indication that the index case had acquired the outbreak strain outside of Norway and the outbreak continued into 2022. Five cases were associated with infection. Detailed investigations of these outbreaks are ongoing.

In addition, minor clusters of carbapenemase-producing *K. pneumoniae* were identified. Two clusters of ST147 isolates: one cluster of two ST147-*bla*_NDM-1_ isolates identified in 2019 previously linked to nosocomial transmission within Norway not linked to import [37] and one cluster identified in 2021 of three isolates harbouring *bla*_NDM-1_ (n = 2) or *bla*_NDM-1_ + *bla*_KPC-2_ without a clear epidemiological link and no association to travel or missing import information. Four out of the five ST147 cases were cases of infection. The genomic and epidemiological data also indicated two separate cases of possible single case onward transmission of *K. pneumoniae* ST392-*bla*_OXA-48_ imported from Spain. These were identified between 2018 and 2019 and in 2020. One of the four ST392 cases was infected. 

## Discussion

In this nationwide study we observed an increasing incidence of CPE cases in Norway with cases near doubling during the study period. The mean incidence rate during 2015–2021 (0.91 per 100,000 person-years) is seven-times that of the 2007–2014 period (0.13 per 100,000 person-years) (Figure 1A) [17]. The increase is concerning and most likely would have been higher if not for the COVID-19-pandemic. Less travel related screening during the pandemic explains the reduction in number of CPE isolates associated with colonisation (Figure 1C). The basis behind the increase in number of CPE isolates associated with infection in 2020 and 2021 is unclear but is in part due to the two outbreaks. During the pandemic an overall decline in numbers of reported cases of notifiable infectious diseases in Norway has been noticed [38]. Few studies report national or population-based incidence of CPE cases, hindering comparisons between countries. In an international context, the mean CPE-incidence rate of 0.91 cases per 100,000 inhabitants in Norway is likely low and reflects the Nordic situation. Sweden reports a mean incidence of 1.4 cases per 100,000 inhabitants in 2015–2020 [39]. In the Survey on Carbapenemase-producing Enterobacteriaceae in Europe (EuSCAPE) study, incidence estimates of carbapenemase-producing *K. pneumoniae/E. coli* per 10,000 hospital admissions were 0.2 in Norway in contrast to > 5 in southern European countries such as Greece and Italy [11]. The relatively low proportion of CPE associated with clinical infections (38%; 149/389) and the identification of only nine bloodstream related infections shows that the burden of invasive CPE infections in Norway is still low. However, the MDR profile (Table) challenges treatment options in each case.

We have previously reported that the CPE epidemiology in Norway is characterised by sporadic cases [40]. Although no interregional spread was observed, cases of healthcare-associated transmission as well as two hospital-related clonal outbreaks indicate a change in the epidemiology. Noteworthy, the proportion of isolates (18%; 70/389) reported as not associated with import were represented by a diversity of genetic backgrounds and carbapenemase variants intermingled in the overall CPE population.

In 2007–2014 only 15% of CPE isolates were identified through screening [17]. The large proportion of CPE identified as colonisation (58%; 226/389) in 2015–2021 is likely due to introduction of revised recommendations for screening of CPE in August 2015. Further, increased awareness and establishment of appropriate diagnostics at the laboratories has likely also contributed. The results support the current broad guidelines for screening before admissions to hospitals. Overall, this underlines the importance of continued vigilance, and that targeted screening is crucial in limiting the spread of CPE.

The high proportion of CPE isolates associated with import (63%; 245/389) and the large diversity of species, carbapenemases and STs likely illustrate the global epidemiology of CPE. OXA-48-like and/or NDM-producing *E. coli*/*K. pneumoniae* are the dominant combinations observed in Asia and Europe [11,41] and 90% (220/245) of the isolates associated to import were linked to travel to those continents. A high proportion of CPE in Norway is represented either by specific carbapenemase-genetic background associations (e.g. OXA-244-producing *E. coli* ST38 and OXA-48-producing *K. pneumoniae* ST392) or emergence of successful carbapenemase-variants such as NDM-5 in a diversity of genetic backgrounds including global high-risk ExPEC and *K. pneumoniae* clones [34,35]. Genomic surveillance is important to track these and decrease the possibility for clonal expansion in Norway.

The emergence of OXA-244 in Norway and the high proportion of OXA-48 variants also challenge current diagnostic screening algorithms in CPE detection [42]. In our study, CPE isolates with OXA-244 and other OXA-48-variants had the lowest MIC for meropenem compared with the other groups of carbapenemases (Figure 3). Most laboratories use disc diffusion as primary AST method. The higher sensitivity of the meropenem disc diffusion breakpoint is likely the reason for identification of OXA-48-like-producers with meropenem MIC below the current MIC breakpoint [43]. Thus, there is also grounds for monitoring the genetic determinants of carbapenemase-production and their phenotypic expression per se to optimise diagnostic CPE-screening.

The study has some limitations, such as the lack of antimicrobial susceptibility data on new antimicrobial agents such as cefiderocol, and the β-lactam-β-lactamase inhibitor combinations meropenem–vaborbactam and imipenem–relebactam. In addition, the susceptibility data were acquired by a broth microdilution method that deviates slightly from the International Organization for Standardization (ISO) standard [44]. Moreover, the travel parameter is associated with uncertainties since there are no clear guidelines for reporting this parameter. The travel parameter includes both travel with and without connection to a healthcare institution abroad and we do not have data on the timeline with respect to the positive CPE sample. The lack of available travel information on 18% of isolates also limits identification of possible sources and transmission chains.

A strength of this study, however, is the national requirement of mandatory reporting of CPE-cases including patient characteristics and submission of suspected CPE-isolates to a reference laboratory from all clinical microbiological laboratories. This ensures national population-based data of confirmed CPE-cases. Nevertheless, the observed incidences should be interpreted with caution as there are relatively few CPE cases.

## Conclusion

The near doubling of CPE-case incidence rates in Norway is associated with multiple factors including travel abroad and import of epidemiological successful high-risk clones and carbapenemase-variants. The relative high proportion of cases (18%; 70/389) not directly related to import underlines the potential importance of unidentified transmission routes. The single hospital and intraregional outbreaks are of concern. The downward incidence rate and travel-associated cases during the COVID-19 pandemic is likely to be reversed as international travel increases. Generic infection control measures and adherence to rectal screening recommendations as well as close monitoring of the molecular CPE epidemiology is of particular importance to avoid further transmission, outbreaks and establishment of CPE in Norway.

## Supplementary Data

13000 SupplementaryMaterial

## Supplementary Data

88000 Supplementary Table1
