# Supplementary material for: Nationwide, population-based observational study of the molecular epidemiology and temporal trend of carbapenemase-producing Enterobacterales in Norway, 2015 to 2021
Source: Euro Surveill. 2023 Jul 6;28(27):2200774. doi: 10.2807/1560-7917.ES.2023.28.27.2200774 (PMC10370044; doi:10.2807/1560-7917.ES.2023.28.27.2200774)
Supplement: SupplementaryMaterial [file 22-00774_SupplementaryMaterial.pdf]

Supplementary Material:

This supplementary material is hosted by Eurosurveillance as supporting information alongside the article Nationwide, population-based observational study of the molecular epidemiology and temporal trend of carbapenemase-producing *Enterobacterales* in Norway, 2015 to 2021, on behalf of the authors, who remain responsible for the accuracy and appropriateness of the content. The same standards for ethics, copyright, attributions and permissions as for the article apply. Supplements are not edited by Eurosurveillance and the journal is not responsible for the maintenance of any links or email addresses provided therein.

Supplementary Table A1. Overview of CPE isolates in Norway 2015-2021. Provided as Excel file.

Supplementary Table A2. Distribution of $bla_{CTX\text{-}M}$ with respect to carbapenemase variant.

Carbapenemase-variant[1]	No. of $bla_{CTX\text{-}M}$ positive isolates	No. of $bla_{CTX\text{-}M}$ negative isolates	Total no. of isolates
OXA-48-like	145	53	198
NDM	77	57	134
KPC	10	13	23
VIM	1	7	8

[1] Isolates co-producing two carbapenemases excluded.
